# Screening of Molecular Virulence Markers in *Staphylococcus aureus* and *Pseudomonas aeruginosa* Strains Isolated from Clinical Infections

**DOI:** 10.3390/ijms11125273

**Published:** 2010-12-21

**Authors:** Ani-Ioana Cotar, Mariana-Carmen Chifiriuc, Sorin Dinu, Marcela Bucur, Carmen Iordache, Otilia Banu, Olguta Dracea, Cristina Larion, Veronica Lazar

**Affiliations:** 1 National Institute for Research in Microbiology and Immunology, Cantacuzino, Spl. Independentei 103, cod 060631, Bucharest 060101, Romania; E-Mails: aniioana@yahoo.com (A.-I.C.); carmeniordache78@yahoo.com (C.I.); olgutza_dracea@yahoo.co.uk (O.D.); larioncristina@yahoo.com (C.L.); 2 Department of Microbiology, Faculty of Biology, University of Bucharest, Aleea Portocalelor 1–3, Bucharest 060101, Romania; E-Mails: bamarcelica@yahoo.com (M.B.); lazar@botanic.unibuc.ro (V.L.); 3 Institute for Cardiovascular Diseases Prof. C.C. Iliescu, Bucharest 060101, Romania; E-Mail: otiliabanu@gmail.com (O.B.)

**Keywords:** Staphylococcus aureus, Pseudomonas aeruginosa, virulence, molecular markers, invasive infections

## Abstract

*Staphylococcus (S.) aureus* and *Pseudomonas (Ps.) aeruginosa* are two of the most frequently opportunistic pathogens isolated in nosocomial infections, responsible for severe infections in immunocompromised hosts. The frequent emergence of antibiotic-resistant *S. aureus* and *Ps. aeruginosa* strains has determined the development of new strategies in order to elucidate the different mechanisms used by these bacteria at different stages of the infectious process, providing the scientists with new procedures for preventing, or at least improving, the control of *S. aureus* and *Ps. aeruginosa* infections. The purpose of this study was to characterize the molecular markers of virulence in *S. aureus* and *Ps. aeruginosa* strains isolated from different clinical specimens. We used multiplex and uniplex PCR assays to detect the genes encoding different cell-wall associated and extracellular virulence factors, in order to evaluate potential associations between the presence of putative virulence genes and the outcome of infections caused by these bacteria. Our results demonstrate that all the studied *S. aureus* and *Ps. aeruginosa* strains synthesize the majority of the investigated virulence determinants, probably responsible for different types of infections.

## 1. Introduction

*S. aureus* is a versatile human pathogen with the ability to cause a large spectrum of human diseases, ranging from skin lesions (abscesses, impetigo) to invasive and more serious infections (osteomyelitis, septic arthritis, pneumonia, endocarditis). The ability of *S. aureus* to cause disease has been attributed to an impressive spectrum of cell-wall-associated (protein A, clumping factors, fibronectin binding proteins, and other adhesive matrix molecules) factors, and extracellular toxins (coagulase, hemolysins, enterotoxins, toxic-shock syndrome toxin 1, exfoliative toxins, and Panton-Valentine leukocidin) as virulence determinants. Excepting the toxemic syndromes determined by toxic-shock syndrome toxin 1 (TSST-1), scalded skin syndrome as a result of epidermolytic toxins activity, and food poisoning as a consequence of ingestion of preformed enterotoxins, the pathogenesis of *S. aureus* is the result of interaction between a variety of host factors and the bacterial virulence determinants [1]. The coordinated expression of *S. aureus* virulence factors is regulated by a complex network including the quorum-sensing system *agr* and the well characterized virulence gene regulators. The specific role and/or importance of a virulence factor in *S. aureus* pathogenesis may vary from one infection type to another. When considering that a high number of *S. aureus* strains are more difficult to be treated due to their multiple resistance to antibiotics, the elucidation of *S. aureus* pathogenesis at the molecular level becomes imperative in the fight against this important human pathogen, for the purpose of finding new therapeutic strategies.

*Ps. aeruginosa* is an opportunistic pathogen responsible for chronic lung infection in cystic fibrosis patients, as well as for nosocomial infections in immunocompromised hosts. This bacterium determines many kinds of infections: urinary tract infections, respiratory system infections, dermatitis, soft tissue infections, bacteremia, bone and joint infections, gastrointestinal infections and a variety of systemic infections, particularly in immunosuppressed patients with severe burns, cancer and AIDS. In patients with cystic fibrosis especially disposed to *Ps. aeruginosa* infections, these bacteria are responsible for high rates of morbidity and mortality [2].

The pathogenesis of *Ps. aeruginosa* infections is multifactorial, as suggested by the large number of cell-associated and extracellular virulence determinants of these bacteria. The first step in *Ps. aeruginosa* infections is the colonization of the altered epithelium. Adherence of *Ps. aeruginosa* to the epithelium is mediated by fimbriae, type 4 pili and flagella [2]. After colonization, *Ps. aeruginosa* produces several extracellular virulence factors (alkaline protease and staphylolytic protease, elastase, protease IV, heat-labile and heat-stable hemolysins, phospholipases C and exotoxins A, S, T, U, Y), responsible for extensive tissue damage, bloodstream invasion, and dissemination [3]. Many of the extracellular virulence factors are controlled by cell-to-cell signaling systems [2]. These regulatory systems enable *Ps. aeruginosa* to synthesize virulence factors in a coordinated, cell-density-dependent manner that can allow the bacteria to surpass/exceed the host defense mechanisms. Interference with the virulence factor production dependent of the cell-to-cell signaling system is a promising therapeutic approach for decreasing the rate of morbidity and mortality caused by *Ps. aeruginosa* colonization and infections [2].

The purpose of this study was to characterize the molecular markers of virulence in *S. aureus* and *Ps. aeruginosa* strains isolated from different clinical specimens, in order to evaluate potential associations between the presence of putative virulence genes and the outcome of infections caused by these bacteria.

## 2. Results and Discussion

The results of phenotypic characterization for the presence of enzymatic virulence factors among the analyzed strains are listed in Tables 1 and 2.

We have detected by PCR analysis 15 virulence genes in *S. aureus*, respectively 7 in *Ps. aeruginosa* strains isolated from infections with different clinical pictures. The results of PCR assays are presented in Figures 1–14.

### 2.1. PCR Assays for *S. aureus* Virulence Genes Detection

The initial step in the pathogenesis of *S. aureus* infection is the attachment of the bacteria to the human cell surfaces and implanted devices. Adhesion of *S. aureus* may be mediated by specific cell-surface proteins, or be a result of interactions between the cell-surface proteins and the host proteins, such as von Willebrand factor, fibronectin, fibrinogen and collagen [4]. By these means, *S. aureus* can adhere directly to eukaryotic cell receptors or, alternatively, can bind to plasma-coated inserted devices. Therefore, *S. aureus* produces two types of adhesins. One set has a characteristic LPXTG motif that anchors the adhesin to the components of the extracellular matrix (ECM); the representatives of this kind of adhesins are grouped in the family named microbial surface components recognizing adhesive matrix molecules (MSCRAMMs). This adhesins family constitutes: protein A, the fibronectin-binding proteins (Fnbps), collagen-binding protein (Cna), elastin-binding protein, clumping factors A and B (ClfA and B). The constituents of the second set of adhesins are noncovalently anchored to the cell surface, including the fibrinogen-binding protein (Fgbp) and coagulase. Fibrinogen is the most abundant host protein in endothelial lesions. Among fibrinogen-binding proteins expressed by *S. aureus* on bacterial cells, there are clumping factors A and B (ClfA, ClfB) responsible for typical *S. aureus* clumping in plasma, promoting both adherence to fibrinogen-coated surfaces *in vitro* as well as endocarditis in experimental animal models *in vivo* [5]. ClfA and ClfB have a similar molecular organization and a high sequence similarity. However, their fibrinogen-binding domain (the A domain), with only 26% structure identity, interacts with different parts of fibrinogen, *i.e.*, the γ-chain for ClfA and both the α- and β-chains for ClfB. Moreover, the *clfA* and *clfB* genes are differently regulated; *clfA* being expressed during bacterial growth, whereas *clfB* only during the early logarithmic phase. This aspect raises the question whether ClfA and ClfB might act in synergy to help the cells to attach more firmly to fibrinogen-coated surfaces during the bacterial growth cycle [5]. The results obtained in experimental models suggest that *S. aureus* strains, that specifically express receptors for fibrinogen and fibronectin, are associated with infective endocarditis, while strains producing receptors for bone sialoprotein, collagen, and fibronectin, are associated with osteomyelitis and arthritis [6].

**Figure 1.**
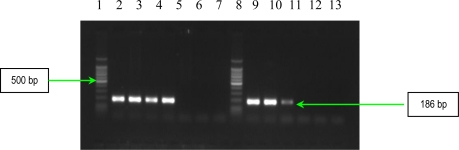
Multiplex PCR assay for simultaneous detection of *bbp* and *ebpS* genes. Lines **1** and **9**:Bench Top PCR Marker (Promega); **2**: *S. aureus* 10936; **3**: *S. aureus* 11372; **4**: *S. aureus* 11327; **5**: MRSA 11325; **6**: negative *S. aureus* strain; **7**: MRSA 11573; **8**: MRSA 11047; **10**: *S. aureus* 5/06; **11**: *S. aureus* 9/06; **12**: *S. aureus* 11323; and **13:** negative control (pure water).

**Figure 2.**
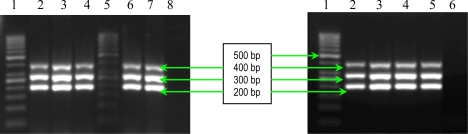
Multiplex PCR assay for simultaneous detection of *fib, clfA*, *clfB* and *fnbB* genes. Left - Lines **1** and **5:** Gene Ruler 50 bp (Fermentas); **2**: *S. aureus* 10936; **3**: *S. aureus* 11372; **4**: *S. aureus* 11327; **6**: MRSA 11325; **7**: MRSA 11573; and **8**: negative control (pure water). Right – Line **1**: Gene Ruler 50 bp (Fermentas); **2**: MRSA 11047; **3:** *S. aureus* 5/06; **4:** *S. aureus* 9/06; **5:** *S. aureus* 11323; and **6:** negative control (pure water).

The results of multiplex PCR1 for detection of *bbp* and *ebpS* genes, showed that *S. aureus* studied strains have no *bbp* gene, while six of them possess the *ebpS* gene (Figure 1). The presence of the elastin binding protein encoding gene in some *S. aureus* analyzed strains, emphasizes their involvement in invasive infections. The PCR2 performed for detection of four MSCRAMMs genes (*fib, clfA*, *clfB* and *fnbB*) showed that all *S. aureus* analyzed strains possess three genes (*fib, clfA*, *clfB)* responsible for encoding fibrinogen-binding protein and clumping factors A and B (Figure 2).

**Figure 3.**
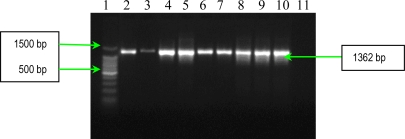
Gel electrophoresis of amplified products of *fnbA* gene. Line **1**: Bench Top 100 bp DNA Lader (Promega); **2**: *S. aureus* 10936; **3**: *S. aureus* 11372; **4:** *S. aureus* 11327; **5**: MRSA 11325; **6**: MRSA 11573; **7**: MRSA 11047; **8**: *S. aureus* 5/06; **9**: *S. aureus* 9/06; **10**: *S. aureus* 11323; and **11**: negative control (pure water).

**Figure 4.**
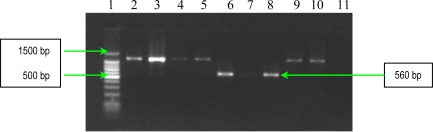
Gel electrophoresis of amplified products of *cna* gene. Line **1**: GeneRuler™ 100 bp Plus DNA Ladder (Fermentas); **2**: *S. aureus* 10936; **3**: *S. aureus* 11372; **4**: *S. aureus* 11327; **5**: MRSA 11325; **6**: MRSA 11573; **7**: MRSA 11047; **8**: *S. aureus* 5/06; **9**: *S. aureus* 9/06; **10**: *S. aureus* 11323; and **11:**negative control (pure water)

The results of PCR analysis concerning the presence of the *fnbA* gene showed that all *S. aureus* strains possess this gene (Figure 3). Fnbps were shown to be required for the internalization *S. aureus* into nonprofessional phagocytic cells. Different studies showed that efficient invasion of *S. aureus* is critically dependent on one of the two known forms of Fnbps, FnbpA or FnbpB. Finally, the results of PCR analysis concerning the presence of *cna* gene, demonstrated the polymophism of this gene among the studied *S. aureus* strains. Seven strains possess a 1120 bp gene, whereas two exhibited a variant of 560 bp gene (Figure 4). The results of PCR assays for adhesins genes showed that all studied *S. aureus* strains possess: elastin-binding protein, clumping factors A and B, fibronectin-binding protein A, fibrinogen-binding protein and collagen-binding protein. The presence of these adhesins demonstrates the involvement of the respective virulence factors in the determined *S. aureus* infections associated with cardiovascular devices.

Bacterial survival during an infection is a process dependent on the ability of the organism to override the host’s defense possibilities. *S. aureus* synthesizes a lot of toxins and exoproteins active against host defense mechanisms. Almost all bacterial strains synthesize a lot of enzymes and cytotoxins including four hemolysins (alpha, beta, gamma, and delta), nucleases, proteases, lipases, hyaluronidase, and collagenase [7]. The main function of these proteins may be to convert local host tissues into nutrients required for bacterial growth. Some strains synthesize one or more additional exoproteins, including toxic shock syndrome toxin-1 (TSST 1), the staphylococcal enterotoxins (SEA, SEB, SEC, SED, SEE, SEG, SEH, and SEI), and the exfoliative toxins A and B [8]. These toxins affect neutrophils and macrophages, gamma-hemolysin, being supplementary, is responsible for the lysis of many varieties of mammalian erythrocytes.

**Figure 5.**
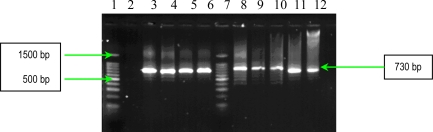
Gel electrophoresis of amplified products of coagulase gene. Lines **1** and **7**: DNA ladder 100bp (Promega); **2**: negative *S. aureus* strain; **3**: *S. aureus* 10936; **4**: *S. aureus* 11372; **5**: *S. aureus* 11327; **6:** MRSA 11325; **8**: MRSA 11573; **9**: MRSA 11047; **10**: *S. aureus* 5/06; **11**: *S. aureus* 9/06; **12**: *S. aureus* 11323; and **13**: negative control (pure water).

Coagulase is an extracellular protein, binding the prothrombin in the host to form a complex called staphylothrombin. The thrombin characteristic protease activity in the complex, determines the conversion of fibrinogen into fibrin. In this way the *S. aureus* cells protect themselves from the phagocytic and immune defense of the host by forming localized clotting. The result of PCR assay shows that all *S. aureus* analyzed strains possessed this factor (Figure 5).

**Figure 6.**
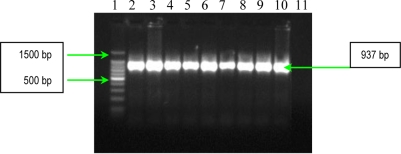
Multiplex PCR assay for simultaneous detection of *luk-PV* si *hlg* genes. Line **1**: DNA ladder 100bp; **2**: *S. aureus* 10936; **3**: *S. aureus* 11372; **4**: *S. aureus* 11327; **5**: MRSA 11325; **6:** MRSA 11573; **7**: MRSA 11047; **8**: *S. aureus* 5/06; **9**: *S. aureus* 9/06; **10**: *S. aureus* 11323; and **11**: negative control (pure water).

Panton-Valentine leukocidin (PVL) is a bicomponent leukocidin responsible for leukocyte destruction and tissue necrosis. PVL, together with gamma-hemolysin, belongs to the recently described family of synergohymenotropic toxins. These toxins determine membrane disruptions of host defense cells and erythrocytes by the synergistic activity of two non-associated classes of secretory proteins, designated as S and F [9]. The results of multiplex PCR for detection of genes encoding these toxins showed that *hlg* gene was present in all analyzed *S. aureus* strains, whereas the *luk-PV* gene was absent (Figure 6). These results are in agreement with those published, that is to say virtually all strains of *S. aureus* synthesize gamma-hemolysin, whereas PVL is synthesized only by less than 5% of strains [10]. However, the PVL gene is frequently seen in *S. aureus* strains associated with necrotic lesions involving the skin, and in severe necrotic hemorrhagic pneumonia, whereas it is seldom seen in strains responsible for other infections, such as infective endocarditis and hospital-acquired staphylococcal infections.

**Figure 7.**
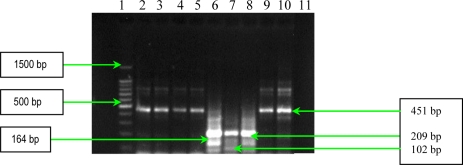
Multiplex PCR assay for simultaneous detection of enterotoxins genes (*sea, seb, sec, sed* and *see*). Line **1**: Bench Top 100bp DNA Lader (Promega); **2**: *S. aureus* 10936; **3**: *S. aureus* 11372; **4**: *S. aureus* 11327; **5**: MRSA 11325; **6**: MRSA 11573; **7**: MRSA 11047; **8**: *S. aureus* 5/06; **9**: *S. aureus* 9/06; **10**: *S. aureus* 11323; and **11**: negative control (pure water).

The results of multiplex PCR assay for detection of staphylococcal enterotoxins genes (*sea, seb, sec, sed* and *see*) showed that four analyzed *S. aureus* strains possess the *sec* gene, whereas two strains possess three genes (*sea, seb* and *see*) (Figure 7). This assay offers a very specific, quick, reliable, and inexpensive alternative to conventional PCR assays used in clinical laboratories to identify various staphylococcal enterotoxin genes.

The variable presence of virulence genes in *S. aureus* strains isolated from blood cultures provides evidence that different bacterial factors play a redundant role in determining invasive disease in hospital units.

### 2.2. PCR Assays for *Ps. aeruginosa* Virulence Genes Detection

*Ps. aeruginosa* exhibits many virulence factors that contribute to its pathogenicity. Some of these factors help colonization, whereas others facilitate bacterial invasion. *Ps. aeruginosa* colonization involves a lot of factors, including fimbriae, type 4 pili, flagella, and surface polysaccharides. The ability of *Ps. aeruginosa* to invade tissues depends upon production of extracellular enzymes and toxins that break down physical barriers by disrupting host cell membranes and annihilating the host, as well as resistance to phagocytosis and host immune defense armamentarium. The tissue invasion by *Ps. aeruginosa* is promoted by the production of proteases, hemolysins and cytotoxin (leukocidin). *Ps. aeruginosa* synthesizes several proteases (LasB elastase, LasA staphylolytic protease, alkaline protease, and protease IV) responsible for: complement inactivation, and surfactant proteins A and D (SP-A and SP-D) degradation. These proteins play an important role in innate immunity, cleavage IgG antibodies, and IFN and TNF inactivation [11]. Elastin is a major part of human lung tissue responsible for lung expansion and contraction. Moreover, elastin is an important component of blood vessels, conferring them their resilience. The concerted activity of LasB elastase and LasA staphylolytic protease is responsible for elastolytic activity, implicated in destruction of elastin-containing human lung tissue and the pulmonary hemorrhages in invasive *Ps. aeruginosa* infections. The LasB elastase is a zinc metalloprotease, encoded by the *lasB* gene, responsible for degradation of elastin, fibrin and collagen [11]. Alkaline protease interferes with fibrin formation, implicated in fibrin lysis, and inactivates important host defense proteins, such as antibodies, complement, IFN-γ, and cytokines. Protease IV is important in the pathogenesis of *P. aeruginosa*-induced microbial keratitis, but little is known about its role in other clinical isolates. The virulence of protease IV in ocular infection has been attributed to the destruction of host proteins, including fibrinogen and components of the immune system. Protease IV also degrades structural proteins, such as elastin, facilitating bacterial adherence and infection [12].

**Figure 8.**
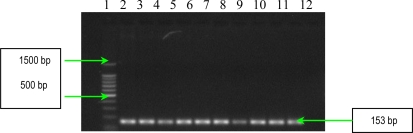
Ethidium bromide-stained 1.5% agarose gel showing the amplified products of *lasB* gene. Line **1**: DNA ladder 100bp (Promega); **2**: *Ps. aeruginosa* 101; **3**: *Ps. aeruginosa* 1558; **4**: *Ps. aeruginosa* 111; **5**: *Ps. aeruginosa* 1443; **6**: *Ps. aeruginosa* 1093; **7**: *Ps. aeruginosa* 1561; **8**: *Ps. aeruginosa* 20; **9**: *Ps. aeruginosa* 1442; **10**: *Ps. aeruginosa* 84; **11**: *Ps. aeruginosa* 1562; and **12**: negative control (pure water).

**Figure 9.**
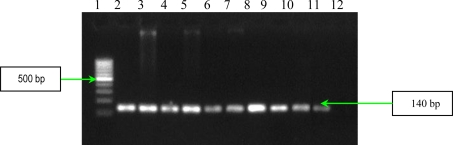
Ethidium bromide-stained 1.5% agarose gel showing the amplified products of *aprA* gene. Lines **1**: Gene Ruler 100bp (Fermentas); **2**: *Ps. aeruginosa* 101; **3**: *Ps. aeruginosa* 1558; **4**: *Ps. aeruginosa* 111; **5**: *Ps. aeruginosa* 1443; **6**: *Ps. aeruginosa* 1093; **7**: *Ps. aeruginosa* 1561; **8**: *Ps. aeruginosa* 20; **9**: *Ps. aeruginosa* 1442; **10**: *Ps. aeruginosa* 84; **11**: *Ps. aeruginosa* 1562; and **12**: negative control (pure water).

**Figure 10.**
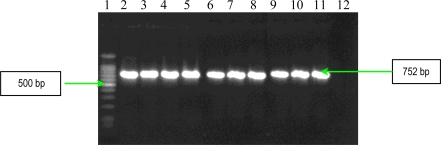
Ethidium bromide-stained 1.5% agarose gel showing the amplified products of *protease IV* gene. Line **1**: DNA ladder 100bp (Promega); **2**: *Ps. aeruginosa* 101; **3**: *Ps. aeruginosa* 1558; **4**: *Ps. aeruginosa* 111; **5**: *Ps. aeruginosa* 1443; **6**: *Ps. aeruginosa* 1093; **7**: *Ps. aeruginosa* 1561; **8**: *Ps. aeruginosa* 20; **9**: *Ps aeruginosa* 1442; **10**: *Ps. aeruginosa* 84; **11**: *Ps. aeruginosa* 1562; and **12**: negative control (pure water).

Results of PCR analysis concerning the presence of genes encoding proteases, showed that all studied *Ps. aeruginosa* strains possess all the tested proteases (LasB elastase, alkaline protease and protease IV), that are correlated with type of infection (Figures 8–10).

**Figure 11.**
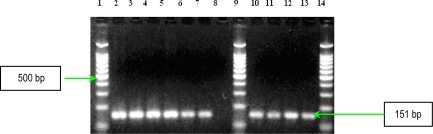
Ethidium bromide-stained 1.5% agarose gel showing the amplified products of *rhlAB* gene. Lines **1**, **9** and **14**: DNA ladder 100bp (Promega); **2**: *Ps. aeruginosa* 101; **3**: *Ps. aeruginosa* 1558; **4**: *Ps. aeruginosa* 111; **5**: *Ps. aeruginosa* 1443; **6:** *Ps. aeruginosa* 1093; **7**: *Ps. aeruginosa* 1561; **8**: negative control (pure water); **10**: *Ps. aeruginosa* 20; **11**: *Ps. aeruginosa* 1442; **12**: *Ps. aeruginosa* 84; and **13**: *Ps. aeruginosa* 1562.

**Figure 12.**
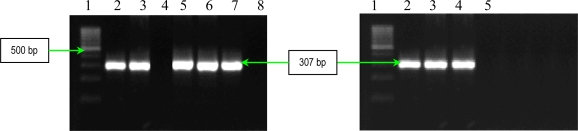
Ethidium bromide-stained 1.5% agarose gel showing the amplified products of *plcH* gene. Left: Line **1**: GeneRuler 100bp DNA ladder (Fermentas); **2**: *Ps. aeruginosa* 101; **3**: *Ps. aeruginosa* 1558; **4**: *Ps. aeruginosa* 111; **5**: *Ps. aeruginosa* 1443; **6**: *Ps. aeruginosa* 1093; **7**: *Ps. aeruginosa* 1561; **8**: *Ps. aeruginosa* 20. Right :Line **1**: GeneRuler 100bp DNA ladder (Fermentas); **2**: *Ps. aeruginosa* 1442; **3**: *Ps. aeruginosa* 84; **4**: *Ps. aeruginosa* 1562; and **5**: negative control (pure water).

**Figure 13.**
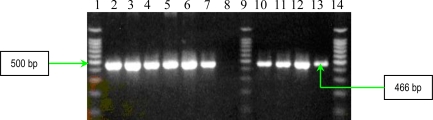
Ethidium bromide-stained 1.5% agarose gel showing the amplified products of *plcN* gene. Lines **1**, **9** and **14**: DNA ladder 100bp (Promega); **2**: *Ps. aeruginosa* 101; **3**: *Ps. aeruginosa* 1558; **4**: *Ps. aeruginosa* 111; **5**: *Ps. aeruginosa* 1443; **6**: *Ps. aeruginosa* 1093; **7**: *Ps. aeruginosa* 1561; **8**: negative control (pure water); **10**: *Ps. aeruginosa* 20; **11**: *Ps. aeruginosa* 1442; **12**: *Ps. aeruginosa* 84; and **13**: *Ps. Aeruginosa* 1562.

**Figure 14.**
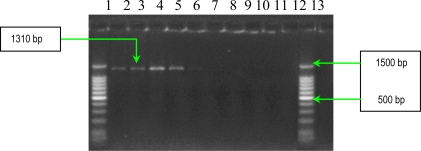
Ethidium bromide-stained 1.5% agarose gel showing the amplified products of *algD* gene. Lines **1** and **12**: DNA ladder 100bp (Promega); **2**: *Ps. aeruginosa* 101; **3**: *Ps. aeruginosa* 1558; **4**: *Ps. aeruginosa* 111; **5**: *Ps. aeruginosa* 1443; **6**: *Ps. aeruginosa* 1093; **7**: negative control (pure water); **8**: *Ps. aeruginosa* 1561; **9**: *Ps. aeruginosa* 20; **10**: *Ps. aeruginosa* 1442; **11**: *Ps. aeruginosa* 84; and **13**: *Ps. aeruginosa* 1562.

Three other soluble proteins involved in *Ps. aeruginosa* invasion are represented by a rhamnolipid, two phospholipases C, haemolytic phospholipase C (PLC-H), and non-haemolytic phospholipase C (PLC-N). These factors may act synergistically to break down phospholipids (e.g., phosphatidylcholine and sphingomyelin), and contribute to invasion by means of their cytotoxic effects on neutrophils, lymphocytes and other eucaryotic cells. Rhamnolipid, a rhamnose-containing glycolipid biosurfactant, has a detergent-like structure and is considered to solubilize the phospholipids of lung surfactant, making them more accessible to cleavage by phospholipase C. The resulting loss of lung surfactant may be responsible for the atelectasis associated with chronic and acute *Ps. aeruginosa* lung infection. Rhamnolipid also inhibits the mucociliary transport and ciliary function of human respiratory epithelium [13]. The results of PCR analysis showed that all *Ps. aeruginosa* possess *rhlAB* gene, encoding the rhamnolipid (Figure 11). Concerning the presence of two phospholipases C, the results of PCR analysis showed that all *Ps. aeruginosa* strains possess *plcN* gene, whereas *plcH* gene is present only in seven of the studied strains (Figures 12 and 13). These two enzymes could work sequentially and synergistically, PLC-H would promote degradation of the erythrocyte membrane (phospholipids components of the outer leaflet: phosphatidylcholine and sphingomyelin), exposing the inner leaflet. PLC-N could then hydrolyze phospatidylserine present in the inner leaflet. The products of phospholipid hydrolysis would be digested by alkaline phosphatase, pyrophosphate being released. The experimental studies demonstrated that purified PLC-H determines vascular permeability, organ injuries, and death when injected in high doses into mice, proving that PLC-H is an important virulence factor. Some *Ps. aeruginosa* strains are producers of a mucoid exopolysaccharide (alginate) that forms the matrix of the *Ps. aeruginosa* biofilm, anchoring the cells and, in certain cases, protecting bacteria from the host defense armamentarium consisting of: lymphocytes, phagocytes, the ciliary action of the respiratory tract, antibodies and complement. At the same time these strains are less susceptible to antibiotics than their planktonic counterparts, being most often isolated from patients with cystic fibrosis and usually found in lung tissues of such individuals. The *algD* gene encodes GDP-mannose dehydrogenase (GMD), which acts as a rate-limiting enzyme in mucoid strains by catalyzing the conversion of GDP-mannose to GDP-mannuronic acid, thereby promoting the cell to alginate production [14]. The results of PCR analysis concerning the presence of *algD* gene showed that only five strains possess this gene, an aspect which demonstrates the involvement of these strains in infections with biofilm formation (Figure 14).

## 3. Experimental Section

### 3.1. Bacterial Strains

In this study nine *S. aureus* and ten *Ps. aeruginosa* strains were studied, selected from a pool of bacterial strains isolated between 2005–2007 from patients hospitalized in Fundeni Hospital, Bucharest. *S. aureus* strains were isolated from patients with infections (blood cultures) with associated cardiovascular devices, whereas *Ps. aeruginosa* strains originated from different clinical specimens (tracheo-bronchical secretions, wound secretions and sputum). The strains identification were performed with the help of conventional and API microtests and VITEK I automatic system.

Concerning the *S. aureus* strains, 23 virulence factors were evaluated, out of which eight (lechitinase, lipase, amylase, caseinase, DN-ase and alpha, beta and delta hemolysins) were assessed using phenotypic tests, and 15 (fibronectin binding proteins A and B, fibrinogen binding protein, clumping factors A and B, elastin binding protein, bone sialoprotein binding protein, collagen-binding protein, Panton-Valentine leukocidin, gamma-hemolysin and SEA, SEB, SEC, SED and SEE enterotoxins) were evaluated using PCR assays.

In the *Ps. aeruginosa* studied strains, 13 virulence factors were evaluated, out of which six (lechitinase, lipase, amylase, caseinase, DN-ase, and hemolysins) were assessed using phenotypic tests, whereas seven (elastase, alkaline protease, protease IV, rhamnolipid, hemolytic phospholipase C, non hemolytic phospholipase C and alginate) were evaluated using PCR assays.

The phenotypic characterization for the presence of enzymatic virulence factors was performed by cultivating the strains in available media for enzymes activity detection [15].

For detection of hemolysins, the strains were streaked on blood agar plates containing 5% (vol/vol) sheep blood in order to obtain isolated colonies. After incubation at 37 °C for 24 h the clear zone (total lysis of red blood cells) around the colonies was registered as positive reaction. Some *S. aureus* strains proved the presence of CAMP-like factor, a special hemolysin, the activity of which is revealed by streaking the bacterial studied strain on 5% sheep blood agar, at 8 mm distance perpendicularly on the β-hemolytic *Staphylococcus aureus* ATCC 25923 reference strain, and incubating the plate aerobically at 37 °C for 24 h. The occurrence of synergic β-haemolysis at the confluence of the two spot areas indicates the presence of CAMP-like factor.

Protease activity was determined using two different media as substrate: 15% soluble casein and starch (final concentration 1% in nutritive gelose) obtained from the Cantacuzino Institute Culture Media Laboratory. The strains were spotted and after incubation for 24 h at 37 °C, precipitation surrounding the growth area indicated casein/starch proteolysis (caseinase/amylase presence) [15].

DNA-se production was studied using DNA agar medium. The strains were spotted and after incubation for 24 h at 37 °C, a drop of HCl/1N solution was added to the spotted cultures; a clearing zone around the culture was registered as a positive reaction [15].

For lecithinase and lipase production, the cultures were spotted onto 2.5% yolk agar, and respectively on Tween 80 agar with a substrate at a final concentration of 1%, and thereafter the plates were incubated at 37 °C up to 7 days. An opaque (precipitation) zone surrounding the spot showed the lecithinase, respectively lipase production [15].

Subsequently, we used multiplex and uniplex PCR assays for the detection of genes encoding the putative’s most important virulence factors in the studied strains (coagulase, adhesins, gamma-hemolysin, enterotoxins for *S. aureus* strains, and elastase, alkaline protease, protease IV, rhamnolipid, alginate, haemolytic phospholipase C and non-hemolytic phospholipase C for *Ps. aeruginosa* strains). Chromosomal DNA was extracted from nineteen clinical strains. One colony of each strain cultured on solid medium was inoculated into 5 mL of BHI (Broth Heart Infusion) and grown overnight at 37°C with shaking. From these strain cultures, DNA extraction was performed by using Wizard DNA Genomic Purification kit (Promega, U.S.) according to the manufacturer′s recommendations. Chromosomal DNAs obtained were used as templates for all PCR experiments. The PCR reactions were carried out in an Applied Biosystems 2700 Thermal Cycler.

### 3.2. Detection of *S. aureus* Virulence Genes by PCR

#### 3.2.1. Multiplex and Uniplex PCRs for Detection of Genes Encoding Adhesins

The detection of **s**taphylococcal microbial surface components recognizing adhesive matrix molecules (MSCRAMMs) with the help of binding assays using purified matrix molecules, is expensive, especially due to the high cost of purified matrix. This phenotypic method has the disadvantage of displaying a redundance of chemical structure of some adhesins: two fibronectin binding proteins (FnbpA and – B) and three receptors for fibrinogen (clumping factors A and B and fibrinogen binding protein, Fib). In addition, some MSCRAMMs bind to more than one matrix molecule (for example, FnbpA binds to both fibronectin and fibrinogen) [6]. In conclusion, the genetic identification of the genes responsible for the respective adhesins synthesis is obviously necessary. PCR assays were used for detection of eight genes encoding MSCRAMMs in *S. aureus* strains isolated from patients with infections associated with cardiovascular devices. The nucleotide sequences of *ebpS* (encoding elastin binding protein), *fnbB* (encoding fibronectin binding protein B), *fib* (encoding fibrinogen binding protein), *clfA* and *clfB* (encoding clumping factors A and B), and *bbp* (encoding bone sialoprotein binding protein) obtained from GenBank (accession numbers U48826, X62992, X72014, Z18852, AJ224764, and Y18653, respectively) were compared and evaluated using Blast and ClustalX softwares to identify regions, unique for each gene, which have similar annealing temperatures [6]. The primers (Invitrogen, U.S.) used for two multiplex PCRs and for two uniplex PCRs, are listed in Table 3, and in Table 4. Two primer sets were prepared for multiplex PCRs: PCR1 to amplify *bbp* and *ebpS* genes, and PCR2 to amplify *fnbB*, *fib*, *clfA*, and *clfB* genes. The thermal cycling conditions for multiplex PCRs included an initial denaturation step (5 min at 94 °C) followed by 25 cycles of amplification (denaturation for 1 min at 94 °C, annealing for 1 min at 55 °C, and extension for 1 min at 72 °C) [6]. The reaction was completed after a 10 min incubation at 72 °C. Synthesized DNA fragments were detected on 1.5% agarose gels by ethidium bromide staining (Figures 1 and 2).

PCR used for detection of *fnbA* gene (encoding fibronectin binding protein A) had the following thermal cycling conditions: an initial denaturation step (5 min at 94 °C) followed by 30 cycles of amplification (denaturation for 1 min at 94 °C, annealing for 1 min at 50 °C, and extension for 2 min at 72 °C) [8]. The reaction was ready after a 10 min incubation at 72°C, and the amplified products were detected on 1.5% agarose gels by ethidium bromide staining (Figure 3).

The detection of *cna* gene (encoding collagen binding protein) was performed by PCR with the specific primers listed in Table 4 [8]. The thermal cycling conditions for *cna* gene amplification included an initial denaturation step (5 min at 94 °C) followed by 30 cycles of amplification (denaturation for 1 min at 94 °C, annealing for 1 min at 55 °C, and extension for 2 min at 72 °C). The reaction was finished after a 10 min incubation at 72 °C and the amplified products were detected on 1.5% agarose gels by ethidium bromide staining (Figure 4).

### 3.3. PCR Assays for Detection of Extracellular Proteins (Coagulase, Panton-Valentine Leukocidin and Gamma–Hemolysin)

Detection of coagulase gene of the staphylococcal studied strains was performed by PCR using the following primers: COAG-2: 5′-CGAGACCAAGATTCAACAAG-3′ and COAG-3: 5′AAAGAAAACCACTCACATCA-3′ [16]. DNA amplification was carried out in thermocycler with the following thermal profile: initial denaturation at 95 °C for 2 min followed by 30 cycles of amplification (denaturation at 95 °C for 30 s, annealing at 58 °C for 2 min, and extension at 72 °C for 4 min) and a final extension at 72 °C for 7 min [16]. Synthesized DNA fragments were detected on 1.5% agarose gels by ethidium bromide staining. A negative control (pure water) was included. The lengths of the PCR products were estimated by comparison with the 100 bp DNA ladder molecular size markers (Promega) (Figure 5).

A multiplex PCR were used for the simultaneous detection of genes encoding the Panton-Valentine leukocidin and gamma-hemolysin. Oligonucleotide primers were designed according to the published sequences of the PVL genes (GenBank accession numbers X72700 and AB006796) and the gamma-hemolysin genes (GenBank accession numbers X81586 and L01055), the former to obtain coamplification of *lukS-PV* and *lukF-PV*, and the latter to obtain coamplification of *hlgC* and *hlgB* [10] (Table 5).

The thermal cycling conditions for *luk-PV* and *hlg* genes co-amplification included an initial denaturation step (5 min at 94 °C) followed by 30 cycles of amplification (30 s of denaturation at 94 °C, 30 s of annealing at 55 °C, and 1 min extension at 72 °C). The reaction was completed with a 10 min incubation step at 72 °C. The PCR products were tested by electrophoresis on 1.5% agarose gels. Synthesized DNA fragments were detected on 1.5% agarose gels by ethidium bromide staining. A negative control (pure water) was included. The lengths of the PCR products were estimated by comparison with the 100 bp DNA ladder molecular size markers (Promega) (Figure 6).

*Multiplex PCR for detection of enterotoxins SEA, SEB, SEC, SED and SEE.* The reaction conditions for the multiplex PCR assay were optimized to ascertain that all of the target gene sequences were satisfactorily amplified. For this purpose, a multiplex set was used containing: 200 mM deoxynucleoside triphosphates; 10 μL of 5X Green GoTaq Flexi Reaction Buffer; 1.5 mM MgCl_2_; 20 pmol (each) of *sea*, *seb*, *sec* and *see* primers; 40 pmol of *sed* primer; 2.5 U of FlexiTaq DNA polymerase (Promega), and 10 to 1,000 ng of template DNA. The volume of this mixture was adjusted to 50 μL with sterile nuclease free water. The primers were designed to target the coding regions of the genes; measures were taken to avoid areas of homology within the enterotoxins structural genes. The primer sequences used in the multiplex PCRs are described in Table 6. DNA amplification was carried out in thermocycler with the following thermal cycling profile: an initial denaturation at 94 °C for 5 min was followed by 35 cycles of amplification (denaturation at 94 °C for 2 min, annealing at 57 °C for 2 min, and extension at 72 °C for 1 min), ending with a final extension at 72 °C for 7 min [17]. The sizes of the amplicons obtained from the studied strains corresponded to the predicted sizes (Table 6). As a negative control, sterile water was used instead of DNA, and no amplicon was observed (Figure 7).

### 3.4. Detection of *Ps. aeruginosa* Virulence Genes by PCR

The prevalence of extracellular virulence genes encoding elastase (*lasB*), alkaline protease (*aprA*), protease IV (TCF), rhamnolipid (*rhlAB*), alginate (*algD*), hemolytic phospholipase C (*plcH*) and non-hemolytic phospholipase C (*plcN*) was determined by PCR assays.

*PCR for detection of elastase, alkaline protease and rhamnolipid genes.* Specific oligonucleotide primers used for detection of genes encoding these virulence factors are listed in Table 7. Parameters for the amplification cycles were: an initial denaturation at 94 °C for 5 min, followed by 30 cycles of amplification (denaturation at 94 °C for 1 min, annealing at 52 °C for 1 min, and primer extension at 72 °C for 1.5 min), ending with a final extension at 72 °C for 5 min [3]. Agarose gel (1.5%) electrophoresis was used for examining the products after PCR (Figures 8, 9 and 11).

PCR for the protease IV gene detection was performed by PCR using specific primers: 5′-TATTTCGCCGACTCCCTGTA-3′ (TCF) and 5′-GAATAGACGCCGCTGAAATC-3′ (TCR) [12]. DNA amplification was carried out in thermocycler with the following thermal cycling profile: an initial denaturation at 94 °C for 5 min was followed by 34 cycles of amplification (denaturation at 94 °C for 30 s, annealing at 60 °C for 30 s, and extension at 72 °C for 2 min), ending with a final extension at 72 °C for 5 min. Synthesized PCR products were detected on 1.5% agarose gels by ethidium bromide staining (Figure 10).

*PCR for alginate, hemolytic phospholipase C and non-hemolytic phospholipase C genes detection*. The genes for these virulence determinants were amplified with primers selected on the basis of the published PAO1 sequence (Table 8) [13]. The amplification protocol was: 94 °C for 3 min, 30 cycles of 94 °C for 30 s, 55 °C for 1 min and 72 °C for 1 min 30 s, and 72 °C for 5 min. Each gene was amplified separately. PCR products were separated in a 1.5% agarose gel for 1 h at 100 V, stained with ethidium bromide and detected by UV transillumination. Amplified genes were identified on the basis of fragment size (Figures 12–14).

## 4. Conclusions

Our study demonstrated that all studied *S. aureus* and *Ps. aeruginosa* strains possess several virulence determinants, many of which are redundant, responsible for the clinical evolution of different infectious processes induced by these infectious agents. All *S. aureus* strains possess seven genes, of which five are encoding adhesins (fibrinogen-binding protein, clumping factors A and B, fibronectin-binding protein A, collagen-binding protein), gamma-hemolysin and coagulase. The *Ps. aeruginosa* strains possess five genes encoding for: LasB elastase, alkaline protease, protease IV, and non-hemolytic phospholipase C. Future studies should be concentrated on validating these findings and the *in vivo* significance of different virulence profiles.

## Figures and Tables

**Table 1.** Soluble virulence factors expressed by *S. aureus* analyzed strains.

**No.**	***S. aureus* strain**	**Source of isolation**	**Lecithinase**	**Lipase**	**Amylase**	**DN-ase**	**Caseinase**	**β-hemolysin**	**Camp factor**
1	10936	Bloodstream culture	++	-	-	++	+++	-	+
2	11372	Bloodstream culture	++	+++	-	+++	+++	++	+
3	11327	Bloodstream culture	++	++	-	+++	+++	-	+
4	MRSA 11325	Bloodstream culture	++	+++	-	+++	+++	++	+
5	MRSA 11573	Bloodstream culture	-	-	-	+++	+++	-	+
6	MRSA 11047	Bloodstream culture	+	-	-	+++	+++	-	+
7	5/06	Bloodstream culture	-	-	-	+++	++	-	+
8	9/06	Bloodstream culture	+±	++	-	+±	+++	+	+
9	11323	Bloodstream culture	++	+++	-	+++	++	++	+

**Table 2.** Soluble virulence factors expressed by *Ps. aeruginosa* analyzed strains.

**No.**	***Ps. aeruginosa strain***	**Source of isolation**	**Lecithinase**	**Lipase**	**Amylase**	**DN-ase**	**Caseinase**	**Hemolysins**
1	101	Tracheo-bronchic secretion	-	++	++	-	-	-
2	1558	Tracheo-bronchic secretion	-	-	+	-	-	-
3	111	Wound secretion	-	+	+	-	++	++
4	1443	Wound secretion	+	-	++	-	+++	++
5	1093	Tracheo-bronchic secretion	++	-	++	-	+++	
6	1561	Wound secretion	++	+	+	-	+++	++
7	20	Sputum	+	+	++	-	++	++
8	1442	Tracheo-bronchic secretion	++	++	+	_	++	++
9	84	Tracheo-bronchic secretion	++	+	-	-	++	-
10	1562	Wound secretion	-	+	-	-	++	+

**Table 3.** Nucleotide sequences and anticipated amplicon sizes for the *S. aureus* gene-specific oligonucleotide primers used in multiplex PCR1 and PCR2.

**The gene**	**Primer**	**Nucleotide sequence**	**Amplicon size (bp)**	**Multiplex PCR**
*bbp*	BBP-1 BBP-2	5′-AACTACATCTAGTACTCAACAACAG-3′ 5′-ATGTGCTTGAATAACACCATCATCT-3′	575	1
*ebpS*	EBP-1 EBP-2	5′-CATCCAGAACCAATCGAAGAC-3′ 5′-CTTAACAGTTACATCATCATGTTTATCTTTG-3′	186	1
*fnbB*	FNBB-1 FNBB-2	5′-GTAACAGCTAATGGTCGAATTGATACT-3′ 5′-CAAGTTCGATAGGAGTACTATGTTC-3′	524	2
*fib*	FIB-1 FIB-2	5′-CTACAACTACAATTGCCGTCAACAG-3′ 5′-GCTCTTGTAAGACCATTTTCTTCAC-3′	404	2
*clfA*	CLFA-1 CLFA-2	5′-ATTGGCGTGGCTTCAGTGCT-3′ 5′-CGTTTCTTCCGTAGTTGCATTTG-3′	292	2
*clfB*	CLFB-1 CLFB-2	5′-ACATCAGTAATAGTAGGGGGCAAC-3′ 5′-TTCGCACTGTTTGTGTTTGCAC-3′	205	2

**Table 4.** Nucleotide sequences of primers used for amplification of *fnbA* and *cna* genes.

**The gene**	**Primer**	**Nucleotide sequence**	**Amplicon size (bp)**
*fnbA*	forward reverse	5′-CACAACCAGCAAATATAG-3′ 5′-CTGTGTGGTAATCAATGTC-3′	1362
*cna*	forward reverse	5′-AGTGGTTACTAATACTG-3′ 5′-CAGGATAGATTGGTTTA-3′.	Multiple of 560 bp

**Table 5.** Nucleotide sequences of primers used for simultaneous amplification of *luk-PV* and *hlg* genes.

**The gene**	**Primer**	**Nucleotide sequence**	**Amplicon size (bp)**
*luk-PV*	*luk-PV-1* *luk-PV-2*	5′-ATCATTAGGTAAAATGTCTGGACATGATCCA-3′ 5′-GCATCAASTGTATTGGATAGCAAAAGC-3′	433
*hlg*	*hlg-1* *hlg-2*	5′-GCCAATCCGTTATTAGAAAATGC-3′ 5′-CCATAGACGTAGCAACGGAT-3′	937

**Table 6.** Nucleotide sequences of primers used for simultaneous amplification of *sea, seb, sec, sed* and *see* genes.

**The gene**	**Primer**	**Nucleotide sequence**	**Amplicon size (bp)**
*sea*	GSEAR-1 GSEAR-2	5′-GGTTATCAATGTGCGGGTGG-3′ 5′-CGGCACTTTTTTCTCTTCGG-3′	102
*seb*	GSEBR-1 GSEBR-2	5′-GTATGGTGGTGTAACTGAGC-3′ 5′-CCAAATAGTGACGAGTTAGG-3′	164
*sec*	GSECR-1 GSECR-2	5′-AGATGAAGTAGTTGATGTGTATGG-3′ 5′-CACACTTTTAGAATCAACCG-3′	451
*sed*	GSEDR-1 GSEDR-2	5′-CCAATAATAGGAGAAAATAAAAG-3′ 5′-ATTGGTATTTTTTTTCGTTC-3′	278
*see*	GSEER-1 GSEER-2	5′-AGGTTTTTTCACAGGTCATCC-3′ 5′-CTTTTTTTTCTTCGGTCAATC-3′	209

**Table 7.** Nucleotide sequences of primers used for amplification of *lasB*, *aprA* and *rhlAB* genes nd anticipated amplicon sizes.

**The gene**	**Primer**	**Nucleotide sequence**	**Amplicon size (bp)**
*lasB*	forward reverse	5′-TTCTACCCGAAGGACTGATAC-3′ 5′-AACACCCATGATCGCAAC-3′	153
*aprA*	forward reverse	5′-ACCCTGTCCTATTCGTTCC-3′ 5′-GATTGCAGCGACAACTTGG-3′	140
*rhlAB*	forward reverse	5′-TCATGGAATTGTCACAACCGC-3′ 5′-ATACGGCAAAATCATGGCAAC-3′	151

**Table 8.** Nucleotide sequences of primers used for amplification of *algD*, *plcH* and *plcN* genes nd anticipated amplicon sizes.

**The gene**	**Primer**	**Nucleotide sequence**	**Amplicon size (bp)**
*algD*	forward reverse	5′-ATGCGAATCAGCATCTTTGGT-3′ 5′-CTACCAGCAGATGCCCTCGGC-3′	1310
*plcH*	forward reverse	5′-GAAGCCATGGGCTACTTCAA-3′ 5′-AGAGTGACGAGGAGCGGTAG-3′	307
*plcN*	forward reverse	5′-GTTATCGCAACCAGCCCTAC-3′ 5′-AGGTCGAACACCTGGAACAC-3′	466
